# Robust profiling of microRNAs and isomiRs in human plasma exosomes across 46 individuals

**DOI:** 10.1038/s41598-019-56593-7

**Published:** 2019-12-27

**Authors:** Tommy A. Karlsen, Tommy F. Aae, Jan E. Brinchmann

**Affiliations:** 1 Department of Immunology, Oslo University Hospital Rikshospitalet, PO Box 4950 Nydalen, 0424 Oslo, Norway; 2 Department of Orthopedic Surgery, Helse Møre and Romsdal HF, Kristiansund Hospital, 6518 Kristiansund, Norway; 3 Institute of Clinical Medicine, Faculty of Medicine, University of Oslo, Oslo, Norway; 4 Department of Molecular Medicine, Faculty of Medicine, University of Oslo, PO Box 1078 Blindern, 0316 Oslo, Norway

**Keywords:** miRNAs, Next-generation sequencing

## Abstract

microRNAs (miRNAs) are small double stranded RNA molecules consisting of two complementary strands called the 5p and 3p arms. Following imprecise processing and/or addition of nucleotides at the ends, miRNA biogenesis can give rise to variants called isomiRs. Exosomes are small vesicles released by cells. They have attracted attention due to their potential use in biomarker development because of their content of biomolecules, including miRNAs and isomiRs. Exosomes are found in body fluids such as plasma. In this study we used next generation sequencing to investigate the distribution of 5p and 3p arms of both miRNAs and isomiRs in plasma exosomes from 46 individuals. Among the canonical miRNAs there was similar prevalence between 5p and 3p miRNAs. Most of the miRNAs had isomiRs, and in approximately half of the cases an isomiR was more abundant than the corresponding canonical miRNA. Most of the isomiRs were generated from 5p miRNAs. There were very small differences in the concentration of canonical miRNA and isomiR sequences between donors, suggesting tight control of isomiR generation and sorting into exosomes. IsomiRs are abundant in plasma exosomes and should be included in analysis when plasma exosomal miRNAs are investigated as potential biomarkers for disease development.

## Introduction

MicroRNAs (miRNAs) are small double-stranded RNA molecules that regulate gene expression. They are involved in most, if not all, biological processes and have been found to be dysregulated in several diseases^1,2^. Monitoring miRNA levels in different cell types, tissues and body fluids, such as plasma, serum and urine has therefore attracted attention because of their potential use as biomarkers for disease development^3^.

During their multistep biogenesis miRNAs are first transcribed as primary transcripts (pri-miRNA). These are processed in the nucleus by the Microprocessor complex, made up by the RNase III enzyme Drosha and two DGCR8 proteins, creating shorter precursor miRNA (pre-miRNA) molecules. The pre-miRNAs are then transported into the cytoplasm where they are further processed, by the RNase III enzyme Dicer, into mature double-stranded miRNA sequences with a length of approximately 20–22 nucleotides (nt). The mature miRNA consists of two sequences, the miRNA-5p (5p) and miRNA-3p (3p) strands, held together by base-pairing and with a 2 nt 3′overhang at each end^2^. After processing by Dicer, one or both of the strands are loaded into the Argonaute (AGO) protein. Here they bind to complementary mRNA molecules leading to either degradation of the mRNA or inhibition of translation^2^. AGO, with the miRNA, mRNA and several proteins mediating mRNA silencing or decay are collectively called the RNA-induced silencing complex (RISC).

miRNAs are defined by their unique sequences as listed in the miRBase data (http://www.mirbase.org/). These sequences are called canonical miRNAs and are defined by the consensus sequence in the database as the most abundant reads obtained from all recorded next generation sequencing (NGS) analysis experiments^4,5^. NGS analysis has revealed several miRNA variations at the ends or within the mature miRNA sequence. These variants are called isomiRs and are thought to be a result of imprecise processing by the Microprosessor complex and/or Dicer or due to addition of nt by nucleotidyl transfereases at the ends of the miRNAs. Another process, RNA editing, can change nt within the miRNA sequence^2^.

The so-called seed sequence, nt 2–8 at the 5′end, is thought to be the most important sequence for binding of the miRNAs to complementary target mRNA sequences, while the 3′end is thought to be important for stabilization of the miRNA^2^. Thus, addition or deletion of nt at the 5′-end will shift the position of the seed sequence and give rise to a new seed with altered target specificity. Substitution of nt within the seed will also give rise to new seed sequences while changes at the 3′-end can affect the stability of the isomiR^2^.

Exosomes are small (40–100 nm) extracellular vesicles that are released from multivesicular bodies (MVB) within the cells into the extracellular milieu. They are formed as intraluminal vesicles within endosomes and contain miRNAs and other non-coding RNAs, mRNA, DNA and proteins^6^. Sorting mechanisms that are not yet fully understood ensure that the exosomal miRNA cargo is different from the overall miRNA content of the parent cell. Exosomes are thought to be involved in intercellular communication since they can be delivered to other cells in the body where they release their content into the recipient cells. During disease the abundance of certain exosomal miRNAs can change^6^. Exosomes from body fluids, such as plasma and serum, has therefore attracted attention due to their potential role in diagnostics or as biomarkers of disease development. Several studies have analysed the content of miRNAs in plasma exosomes^6–8^. However, the distribution of 5p and 3p strands and their corresponding isomiRs in plasma exosomes is not sufficiently described and could be of importance in diagnostics.

In this study we have sequenced and characterized the content of canonical miRNAs and isomiRs in plasma exosomes from 46 individuals. 177 canonical miRNAs and 1716 isomiRs were detected. Both the 5p and 3p strands from the same miRNA were detected in 32% of the canonical miRNA sequences, only the 5p for 36% and only the 3p for 29% of the sequences. 67% of the canonical miRNA had isomiRs while 13% did not. The remaining 20% of the sequences were isomiR sequences where no belonging canonical sequences were detected. In 52% of the cases an isomiR was more abundant than the corresponding canonical miRNA. There was remarkably little difference in the concentration of canonical miRNAs and isomiRs between the donors, suggesting tight control both of the synthesis within the cells and of the release of these sequences into exosomes. IsomiRs are abundant in plasma exosomes and should be considered in biomarker analysis.

## Results

**Figure 1:**
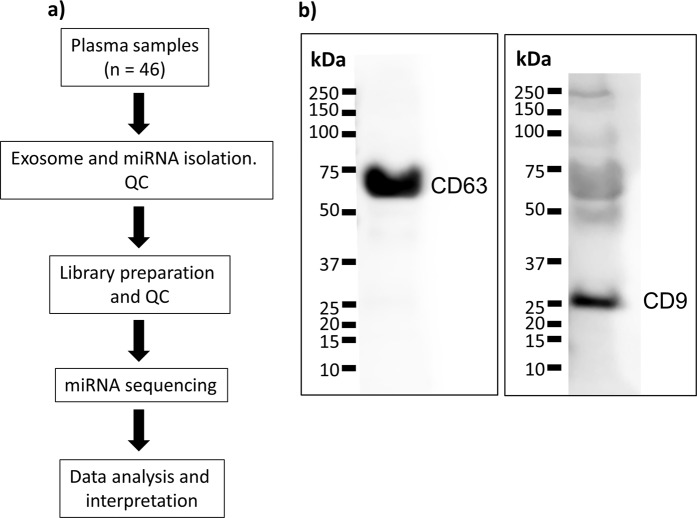
Set up. (**a**) Schematic diagram of the experiment. (**b**) Western blotting of CD63 and CD9 in plasma exosome lysate. The blots are from two different gels and cropped to fit into one figure. Different exposure times were used for the two blots. Unprocessed images of the blots are shown in Supplementary Fig. S1.

A flowchart of the experiment is shown in Fig. 1a. Western blot of the tetraspanins CD63 and CD9, found in the membrane of exosomes, confirmed isolation of exosomes (Fig. 1b). An unprocessed image of the blot is shown in Supplementary Fig. S1.

### Distribution of canonical 5p and 3p sequences

**Figure 2:**
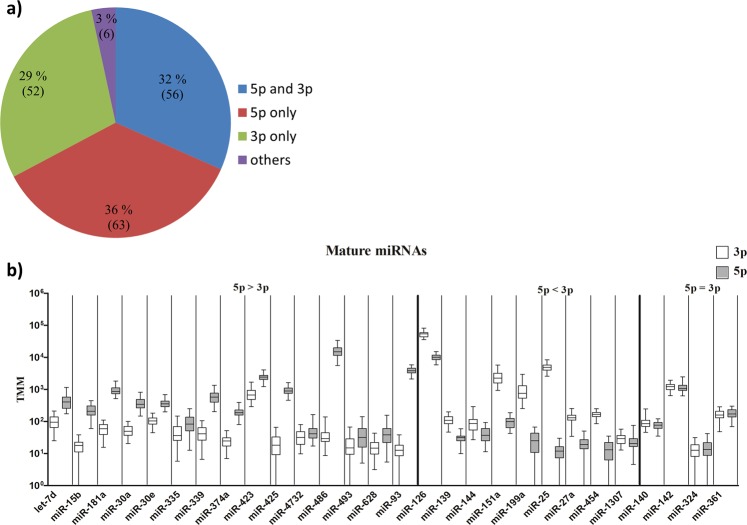
Distribution of 5p and 3p sequences. (**a**) Canonical miRNA sequences as deriving from 5p strands, 3p strands or from both strands of a miRNA. (**b**) Expression levels of canonical 5p and 3p among the 28 miRNA pairs where both strands were represented shown as boxplots with median, 75^th^% and 25^th^% percentiles and the minimum and maximum values. A paired t-test (p = 0.05) was used to test for differences. 5p = 3p represent non-significant results. TMM = Trimmed mean of M-values normalization.

Supplementary Table S1 shows the names of all detected miRNAs and isomiRs, their sequences and TMM (Trimmed mean of M-values normalization) expression values for all samples. In total 1893 sequences were detected. Canonical miRNA sequences constituted 177 of these sequences while 1716 sequences were isomiRs. The abundance of canonical 5p and 3p sequences is shown in Fig. 2a. 32% of the canonical miRNAs sequences were 5p and 3p strands from the same miRNA (28 pairs), while for 36% and 29% of the miRNAs only the 5p or the 3p sequences was detected, respectively. Canonical miRNAs without a 5p or 3p annotation constituted 3% of the sequences. Figure 2b shows which of the two arms were the most prevalent among the 28 miRNAs where both strands were detected. 5p strands were more prevalent than 3p in 54% of the pairs (15 of 28), 3p more prevalent than 5p in 32% of the pairs (9 of 28) and equal prevalence was detected in 14% of the pairs (4 of 28). Overall, canonical 3p sequences were found to be as numerous as 5p sequences in plasma exosomes.

### Distribution of canonical and isomiR sequences

**Figure 3:**
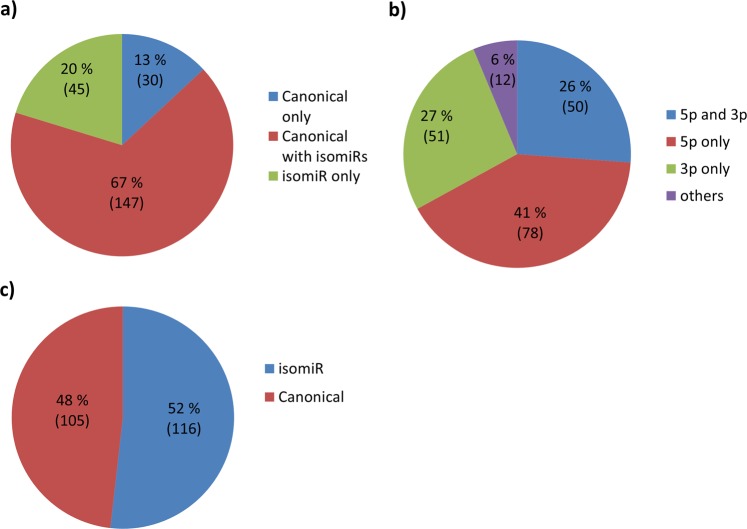
Canonical miRNA and isomiRs. (**a**) Distribution of canonical miRNA and isomiRs. Some miRNAs were represented by canonical sequences only (blue), some by both canonical sequences and isomiRs (red) and som by isomiR sequences only (green). (**b**) Distribution of isomiRs derived from 5p strands (red), 3p strands (green) or from both strands of a miRNA (blue). (**c**) Pie chart showing the proportion of miRNAs where the canonical sequence was most abundant (red) or an isomiR sequence was most abundant (red).

The number of exosome isomiRs per canonical miRNA varied hugely, from 0 to 103. The distribution of the sequences is shown in Fig. 3a. Canonical miRNAs without corresponding isomiR sequences constituted 13% of all sequences, while 67% of the sequences were miRNAs with corresponding isomiRs. IsomiRs without corresponding canonical miRNA sequences made up the remaining 20%. 26% of the isomiRs were variants of either strand of miRNAs where both 5p and 3p isomiRs were detected, 41% were from 5p sequences only, 27% were 3p sequences only and 6% were from miRNA without the 5p/3p annotation (Fig. 3b). In 52% of the cases at least one isomiR was more abundant than its corresponding canonical miRNA sequence (Fig. 3c). However, only 6.3% of the more abundant isomiRs contained new seed sequences. On the whole, many more isomiRs than canonical miRNAs were found in the exosomes and most isomiRs were from the 5p arm.

### Stable miRNA and isomiRs concentration in plasma exosomes from different donors

**Figure 4:**
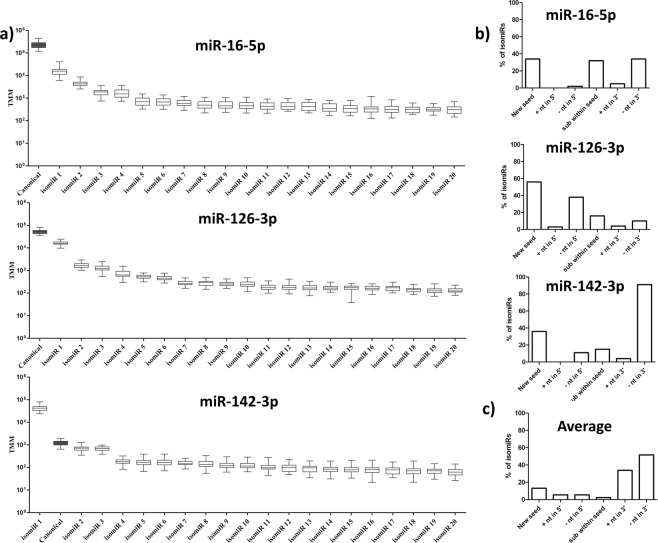
miRNA and isomiR expression. (**a**) Expression levels of canonical miR-16-5p, miR-126-3p, miR-142-3p (grey boxplots) and their top 20 isomiRs (white boxplots). TMM = Trimmed mean of M-values normalization. (**b**) Distribution of changes at the 5′-end, within the seed sequence and at the 3′-end of isomiRs for miR-16-5p, miR-126-3p and miR-142-3p. (**c**) Average distribution of changes at the 5′-end, within the seed sequence and at the 3′-end of all isomiRs. +nt in 5′ = addition of nucleotides at the 5′end, −nt in 5′ = removal of nucleotides at the 5′-end, sub within the seed = substitution of nucleotides within the seed sequence, +nt in 3′ = addition of nucleotides at the 3′end, −nt in 3′ = removal of nucleotides at the 3′-end.

The three most abundant sequences were miR-16-5p, miR-126-3p and a miR-142-3p isomiR. They are plotted, showing the 20 most abundant sequences, in Fig. 4a. miR-142-3p is an example where an isomiR sequence was more abundant than the canonical miRNA. There were surprisingly small inter-donor differences as shown by the very low 75–25 percentile boxes and the maximum and minimum values in the boxplots. Also for the least abundant sequences, such as miR-628-3p, the inter-donor differences were small for both canonical miRNAs and isomiRs (Supplementary Fig. S2).

### New seed sequences in isomiRs

IsomiRs differ from canonical sequences at the 5′end, 3′end or within the sequence. Addition or removal of nt at the 5′-end and changes within the seed sequence will give rise to new seed sequences and therefore new mRNA targets, while changes at the 3′-end can affect stability^2^. The distribution of changes at the 5′end, within the seed and at the 3′-end varied a lot between different miRNAs. Figure 4b shows the fraction of isomiRs with changes leading to new seed sequences as well as changes at the 3′-end as a percentage of all isomiRs for the three most abundant miRNAs sequences shown in Fig. 4a, while Fig. 4c shows the fraction changes as the average percentages of all isomiRs found in plasma exosomes. On average 13% of all isomiRs contained new seed sequences, equally divided at 5.5% each between additions and deletions of nt at the 5′ end, with substitutions within the seed sequence found for approximately 2% of all isomiRs. However, when the TMM for sequences with new seeds was calculated as a percentage of the TMM for all identified sequences, most of these new seed sequences showed very low abundance. Table 1 shows the percentage concentration of new seed sequences among the 10 most abundant miRNAs measured as TMM relative to the TMM for all the isomiR sequences within that miRNA. Except for miR-126-3p and miR-142-3p, isomiRs with new seed sequences always represented <2% of all sequences.

**Table 1:** Percentage of average total TMM for top 10 expressed sequences.

	% new seed	% canonical seed
hsa-miR-16-5p	2.0	98.0
hsa-miR-126-3p	24.9	75.1
hsa-miR-142-3p	4.6	95.4
hsa-let-7a-5p	1.2	98.8
hsa-let-7f-5p	1.0	99.0
hsa-let-7b-5p	0.7	99.3
hsa-miR-486-5p	1.0	99.0
hsa-let-7i-5p	0.6	99.4
hsa-miR-451a	1.2	98.8
hsa-miR-92a-3p	1.2	98.8

98.5% of all isomiRs had changes at the 3′ end. Loss of nt was more common than addition of nt at the 3′-end (Fig. 4c).

**Figure 5:**
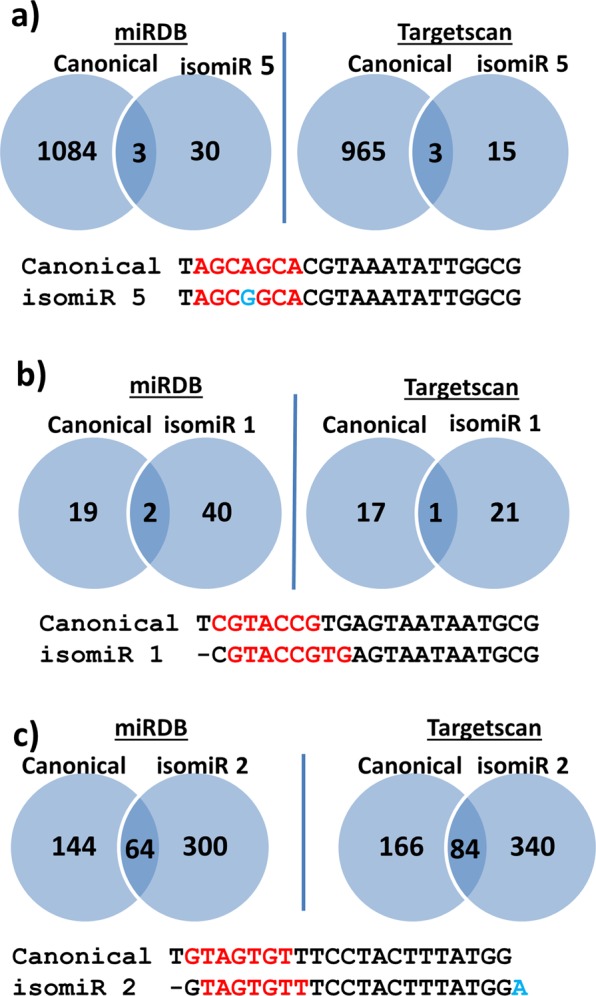
Target prediction. (**a**) mRNA target prediction of miR-16-5p and its isomiR 5 from Supplementary Table S1. The canonical miRNA and isomiR sequences are shown at the bottom with the seed in red and the nucleotide substitution in blue. (**b**) mRNA target prediction of miR-126-3p and its isomiR 1 from Supplementary Table S1 (**c**) mRNA target prediction of miR-142-3p and its isomiR 2 from Supplementary Table S1. The canonical and isomiR sequences are shown at the bottom with the seed in red and addition of a nucleotide at the 3′-end in blue. Numbers are the total number of mRNA targets predicted by each of the databases.

Using two target prediction databases, miRDB and Targetscan, to predict mRNA targets for the different miRNA sequences showed huge differences in the number of targets when comparing canonical miR-16-5p, miR126-3p and miR-142-3p with the most abundant of their isomiRs with new seed sequence (Fig. 5a–c). For the miR-16-5p isomiR 5 the new seed was a result of substitution of nucleotide number 5 (A/G). This resulted in a dramatic loss of predicted targets and only 3 targets were common between the canonical and the isomiR (Fig. 5a). For miR-126-3p the isomiR had more predicted targets than the canonical sequence, but only 1 and 2 targets were in common between the canonical and the isomiR in the two databases (Fig. 5b). Loss of one nucleotide at the 5′-end of miR-142-3p resulted in a new seed sequence that doubled the number of predicted targets and more than 60 targets were common between the canonical and the isomiR (Fig. 5c).

## Discussion

Exosomes carry proteins and nucleic acids, including miRNAs and their isomiRs^6^. The RNA content within exosomes is protected against degradation by RNases and can therefore be isolated intact from body fluids^9^. Stability and availability make miRNAs and isomiRs promising as biomarkers. Although isomiRs have been studied in plasma and other tissues^10,11^, to the best of our knowledge, no studies have investigated the abundance and distribution of isomiRs in plasma exosomes. The data in this study presents the distribution and abundance of canonical 5p and 3p arms and canonical miRNAs and isomiR sequences in plasma-derived exosomes.

It is not yet fully known how the selection of miRNAs for incorporation into exosomes occurs. One possibility is that miRNAs are transported by RNA-binding proteins (RBP) from the RISC to MVBs for exosome loading. However, it is also possible that the strand not incorporated into RISC is carried by RBP to the MVBs. One mechanism for sorting of miRNA sequences bound for exosomes is therefore at the level of binding to AGO. Which of the two strands, 5p or 3p, that are incorporated into the RISC complex depends on the thermodynamic stability of the strands and the identity of the 5′-terminal nucleotide^2^. The term “guide strand” has been used to name the active strand that is incorporated into RISC while “passenger strand” has been used to name the opposite and degraded strand^12^. Historically the 5p strand was thought to predominate as the guide strand^12^. However, while the correlation of their presence in exosomes and their binding to AGO is not yet known, the similarity in the prevalence of 5p and 3p strands in exosomes found here suggests that “guide” and “passenger” terminology for the 5′ and 3′ strands, respectively, is not helpful for our understanding of the mechanisms involved in the sorting of miRNAs bound for exosomes. Another sorting mechanism could be at the level of binding to RBP. This has already been shown for the RBP heterogeneous nuclear ribonucleoprotein A2B1, where both sequence and sumoylation are determinants of miRNA binding^13^. Yet another factor affecting the prevalence of miRNAs in plasma exosomes is which cells are actually contributing miRNA-containing exosomes to the plasma pool. Here very little is known at the present time. Extreme possibilities are that each contributing cell type is responsible for all the copies of one or a few miRNAs, which is unlikely based on the heterogeneity found in miRNAs in exosomes derived for instance from synovial fluid or cell culture supernatants^14,15^, or that every contributing cell type release exosomes containing most or all of the miRNA sequences described in this study. However, whichever mechanisms act in the regulation of miRNA sequences present in the plasma exosome cargo they seem to exert tight control, as suggested by the relatively low number of canonical miRNA sequences found – 177 out of a total of 1917 miRNAs found in miRbase – and the minimal difference in the prevalence of the different miRNA sequences observed between the 46 plasma donors studied here. Interestingly, canonical 3p miRNAs were as prevalent as 5p miRNAs in plasma exosomes, an observation which was also made following analysis of miRNA sequences in a very large number of human tissues^16^.

The vast majority of canonical miRNAs found in plasma exosomes in this study were present together with one or more isomiRs. This is comparable with other studies on cancer cells and plasma^17–19^. In approximately half of the cases one isomiR, or several, was more prevalent than the corresponding canonical miRNA. This is similar to observations made in cultured chondrocytes, where only half of the top 20 expressed miRNAs had the canonical miRNA as the major expressed sequence^20^. Thus, isomiRs seem to be very common and highly expressed in many tissues and they probably have important roles in gene regulation. This is supported by studies were it has been demonstrated that isomiRs do indeed have a functional role^21^. isomiRs can have a different seed than its corresponding canonical miRNA and thus have different mRNA targets. New seed sequences were found in 13% of all the isomiR sequences. This was equally a result of removal and addition of nt at the 5′-end, with substitution within the seed occurring less commonly. In silico analysis showed that there were huge differences in the number of predicted targets and few shared genes between the canonical miRNAs and the isomiRs. However, it should be noted that isomiRs with new seeds made up a very small fraction, compared to sequences with the canonical seed,when taking expression level into account. The importance of plasma exosomes in cell communication is not well known, but *in vitro* and *in vivo* studies have shown that exosomes can be delivered and taken up by recipient cells and affect gene expression^6^. Thus, both for their roles in intercellular communication and also for their use as biomarkers for disease one would think, and hope, that changes in the miRNA cargo in exosomes released from cells in sick organs are sufficient in magnitude that distant cells, and investigating laboratories, will detect that change within the content of plasma exosomes. Based on current knowledge, that change may occur both in canonical miRNA and in isomiR sequences. For profiling and functional studies it is therefore important to include isomiRs in the analysis.

PCR based assays, microarrays and northern blots do normally not discriminate between highly similar sequences. Consequently, many miRNA profiling studies have measured not only the canonical miRNAs, but also one or several of their isomiR sequences. As isomiRs are now known to be at least as prevalent as their canonical miRNAs, this suggest that miRNA quantification studies published using these assays may have based their conclusions on incorrect data. For functional studies, when cells are transfected with pre-made miRNA mimics, the sequences are based on the canonical miRNA sequence in miRbase. If the main functionality of the miRNA in question is exerted by an isomiR, these miRNA mimics are unlikely to reproduce that functionality. However, by using custom-made mimics it is possible to study the functionality of both canonical miRNA and their isomiRs. Transfection of plasmids or viral vectors with the miRNA gene, on the other hand, will presumably be processed by the Microprocessor complex and Dicer and thus give rise to isomiRs. However, whether the generated isomiRs are similar to the endogenous isomiR pool is unknown. This strategy may give correct information about the functionality of the processed pri/pre-miR, but not of the canonical miRNA or individual isomiR sequences.

We conclude that the release of miRNA and isomiRs into exosomes seems to be a tightly regulated process. Surprisingly the 3p strand was found to be as prevalent as the 5′ strand in plasma exosomes and both strands were associated with isomiRs. IsomiR analyses should be included when biomarker studies are being planned.

## Methods and Materials

### Collection of plasma and storage

Blood from 46 individuals was collected in EDTA tubes. The donors were recruited from the Musculoskeletal pain in Ullensaker Study (MUST)^22^ as 23 patients with osteoarthritis (OA) (7 males and 16 females; mean age 58 years, range 45–72; mean body mass index 27.6, range 23.5–39.2) and their age, gender and body weight matched controls without OA (7 males and 16 females; mean age 57.7 years, range 42–72; mean body mass index 27.1, range 22.3–38.4) in a study to look for a miRNA biomarker for OA in plasma exosomes. However, a paired differential expression analysis, using a quasi-likelihood F-test, showed no difference between patients and controls in the expression of either canonical miRNAs or isomiRs after multiple hypothesis testing (false discovery rate was above 0.99 for all sequences). A paper describing patient characteristics, the expression of OA related canonical miRNAs and the 20 canonical miRNAs with the highest expression has been submitted for publication. In the present analysis of all canonical miRNAs and isomiRs found in plasma exosomes the patients and controls are analysed as one group, as no difference in expression was found between the diagnostic subgroups. All donors signed a written informed consent. The study, including all methods and experiments, was approved by the Regional Committee for Medical Research Ethics, Southern Norway, Section A. The study was performed in accordance with the ethical standards of the Helsinki Declaration of 1975, as revised in 2000.

After centrifugation the plasma was stored at −80° Celsius until exosome isolation and analysis. Exosome isolation and miRNA sequencing were performed using Qiagen services (Qiagen, Vedbaek, Denmark). Exosome miRNAs were isolated using the exoRNeasy Serum Plasma Kit according to the protocol from the manufacturer (Qiagen). This is a column based kit where exosomes are captured on a membrane and allows for removal of contaminants such as plasma proteins and protein/AGO-bound miRNAs^23^.

### Library preparation and NGS

The library preparation was done using the QIAseq miRNA Library Kit (Qiagen). Adapters containing unique molecular identifiers were ligated to the RNA before conversion to cDNA. After PCR (22 cycles), the samples were purified. Library preparation quality control was performed using either Bioanalyzer 2100 (Agilent, Santa Clara, California, United States) or TapeStation 4200 (Agilent). The libraries were pooled in equimolar ratios and sequenced on a NextSeq500 machine as single-end reads (51 nucleotides) with an average depth of 22 million reads per sample. FASTQ files were generated using the bcl2fastq software (Illumina Inc., San Diego, California, United States) and checked using the FastQC tool. The reads were mapped to the GRCh37 reference genome using Bowtie2 (2.2.2). Reads were normalised using trimmed mean of M-values (TMM) normalization. Filtering on sequencing depth normalized values was applied before accounting for composition bias. A threshold of 12 counts per million reads (CPMs) in at least half the number of samples was used for inclusion of miRNAs in the analysis.

IsomiRs were identified as follows: analysis was performed individually for each sample based on the occurrence of count variants for each detected microRNA. Reads were mapped to known microRNAs according to the annotation in miRBase and then investigated for the presence of different isomiRs. These variants were identified by changes in start or stop position, or occurrence of mutations within the read. The results for each sample were then merged to generate a single count file with a consistent nomenclature across the samples. Only isomirs that were present at a level of 5% of total reads for that miRNA were retained.

IsomiRs were identified as such, and not novel miRNAs, because they mapped to miRbase version 20 miRNA reads with less than perfect matching. Reads with variations to the miRbase reference, such as mismatch and alternative start/end positions, were reported as isomiR counts. Novel miRNAs would not map to miRbase reads, but would map to the genome at loci that do not encode known miRNAs.

The study complied with the Minimum Information about a Microarray Experiment (MIAME) checklist. The sequencing data are available from the corresponding author on reasonable request.

### Western blotting

Exosomes were isolated using the exoRNeasy Serum Plasma Kit and eluted in 100 µl Buffer XE (Qiagen), mixed with 100 µl of 2x Laemmli Sample Buffer (Sigma-Aldrich, St. Louis, MO), vortexed for 10 seconds and incubated at 98 °C for 10 minutes to denature proteins. 35 µl lysate was loaded onto a 10% polyacrylamide gel (Biorad, Hercules, CA). Proteins were separated by gel electrophoresis, transferred to PVDF membranes using the TransBlot Turbo system (Biorad) and incubated with mouse anti-human CD9 (Abcam, Cambridge, UK) and mouse anti-human CD63 (Abcam) antibodies. After washing, incubation with a horseradish peroxidase-conjugated horse anti-mouse IgG (H + L) secondary antibody (Vector labs, Burlingame, CA) and a final washing step the bands were visualized using the myECL imager (Thermo Fisher Scientific, Waltham, MA). All antibodies were diluted in 1X TBS, 5% nonfat dry milk, 0.1% Tween 20.

## Supplementary information

Supplementary Figures

Supplementary Table S1
