# Supplementary material for: Robust profiling of microRNAs and isomiRs in human plasma exosomes across 46 individuals
Source: Sci Rep. 2019 Dec 27;9:19999. doi: 10.1038/s41598-019-56593-7 (PMC6934752; doi:10.1038/s41598-019-56593-7)
Supplement: Supplementary file 1 — Supplementary Figures [file 41598_2019_56593_MOESM1_ESM.pdf]

Supplementary Figures

Robust profiling of microRNAs and isomiRs in human plasma exosomes across 46 individuals

Tommy A. Karlsen[1*], Tommy F. Aae[2] Jan E. Brinchmann[1,3]

[1]Department of Immunology, Oslo University Hospital Rikshospitalet, PO Box 4950 Nydalen, 0424 Oslo, Norway

[2] Department of Orthopedic Surgery, Kristiansund Hospital, 6518, Kristiansund, Norway.

[3]Department of Molecular Medicine, Faculty of Medicine, University of Oslo, PO Box 1078 Blindern, 0316 Oslo, Norway

Supplementary Fig. S1.

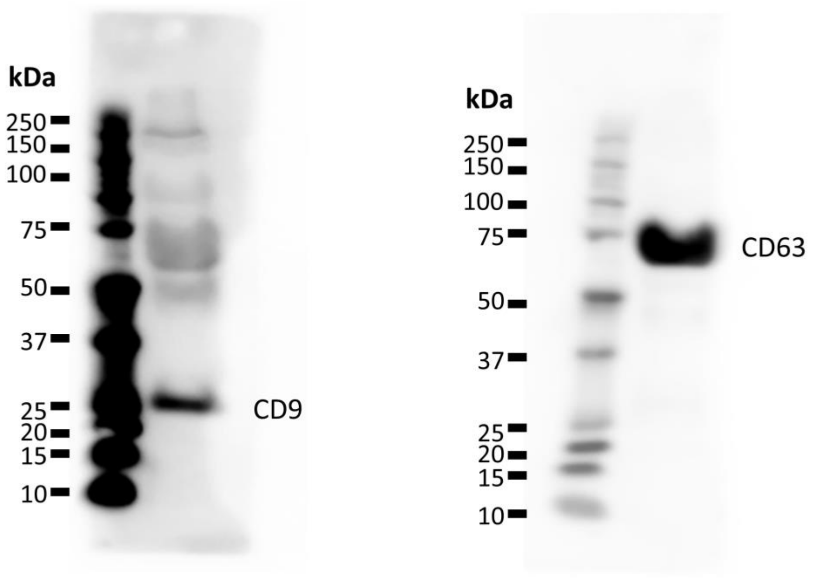

Supplementary Fig. S1. Unprocessed images of western blots of CD9 and CD63. Size markers for the ladder are inserted at the left side of each blot.

Supplementary Fig. S2

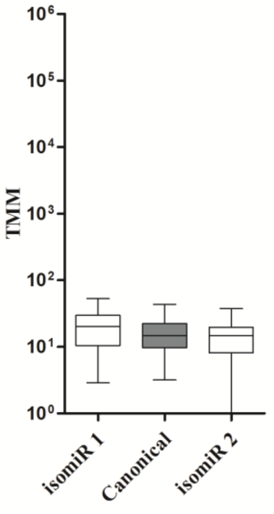

Supplementary Fig. S2. Expression levels of canonical miR-628-3p and two isomiRs: isomiR 1 and isomiR 2. TMM = Trimmed mean of M-values normalization.
